# Surface-energy ratchet motor with geometrical symmetry driven by biased random walk

**DOI:** 10.1038/s41598-024-67383-1

**Published:** 2024-07-18

**Authors:** Miku Hatatani, Daigo Yamamoto, Akihisa Shioi

**Affiliations:** Department of Chemical Engineering and Materials Science, Doshisha University, 1-3 Tatara Miyakodani, Kyotanabe, Kyoto 610-0321 Japan

**Keywords:** Chemical physics, Statistical physics, thermodynamics and nonlinear dynamics

## Abstract

A geometrically symmetric gear with asymmetric surface wettability exhibits one-way spin on a vibrating water bed. On the side face of the gear, a parafilm was coated to create asymmetry in the surface energy. The gear shows fluctuations in both directions within a shorter timescale; however, for a longer timescale, the gear exhibits a one-way spin. This unique motion is generated by a stochastic process with a biased driving force produced by the interaction between the vibrating water surface and the side face of the gear. This new model resembles an active Brownian ratchet. Until now, most ratchet motors, which obtain regular motion from nonthermal fluctuations, utilize a geometrical ratchet structure. However, in this study, the surface energy forms a ratchet that rectifies the noisy motion.

## Introduction

The spontaneous rectification of noisy motion to a regulated form using a ratchet mechanism is a fascinating energy-conversion system^1–4^. In biological systems, a Brownian ratchet has been proposed to understand the mechanism of molecular motors, where the periodically varying potential produced by a chemical reaction is considered to rectify the thermal motion of molecules to generate net transport^5–13^. The pattern of the potential field periodically changes between uniform and ratchet shapes, resulting in directed transport. Thus, rectification generates mechanical work. The efficiency of energy conversion is reported to be higher in vivo than in vitro^14–16^. Physiological chemical reactions in vivo modulate thermal motion to generate nonthermal fluctuations, which may be crucial for the ratchet mechanism. Energy conversion from nonthermal agitation to a mechanically utilized form has attracted significant attention in the field of nonlinear science^1–3^. This scientific trend has inspired ratchet motor studies that extract regulated motions from nonequilibrium fluctuations.

According to the second law of thermodynamics, uniform thermal fluctuation cannot generate regular motion spontaneously, whereas fluctuations in the nonequilibrium state can generate mechanical work. Although random and noisy motions in a nonequilibrium state should be distinguished from thermal motion, various types of nonequilibrium noisy motions exist^17–19^. Some may be close to thermal motion, whereas others may be far from it. The types of noisy motions that can be rectified by a proper ratchet mechanism are an intriguing and fundamental question in science. This understanding will facilitate the development of energy-harvesting technologies and advancements in biological science. From this viewpoint, many scientists have attempted to produce visible models of a ratchet motor^20–24^. The Feynman–Smoluchowski ratchet is the one of the most classical systems^25–28^. The random collision of particles to a paddle wheel generates a random fluctuation in wheel rotation. However, a pawl prevents motion in one of the possible directions; hence, the wheel exhibits directed spin. This idea has led to the progress of ratchet-motor studies, and most ratchet systems use a geometrically asymmetric ratchet to rectify nonthermal fluctuations^17–19,29–33^. In these studies, mechanical vibrations were occasionally employed as a source of agitation. In most cases, mechanical agitation is sinusoidal. However, this can induce complicated and nearly random agitation in vibrating media. Numerous studies utilizing mechanical vibrations have been conducted for ratchet motor systems^34,35^. For example, a circular ratchet system with an inner sawtooth-shaped core^36–38^, droplet moving in one direction on a vibrating asymmetrically structured substrate^39–41^, and spinning “vibrot” on a vibrating substrate^42–44^ were studied. These studies address the conversion from vertical agitation to directed motion, where the ratchet mechanism is attributable to the geometrical asymmetry of the ratchet.

The authors have also reported a geometrical ratchet gear on a vibrating medium^45,46^. The idea of this system originated from studies based on the Feynman–Smoluchowski ratchet; however, the revealed mechanism was completely different. In the vibrating water and granular beds, the water and granules exhibited symmetrical motions along the circumferential direction. The produced one-way spin was not produced by the pawl mechanism (selective inhibition of possible motions). Instead, the system generated torque, leading to one-way spin from a circumferentially symmetric fluctuation. This was particularly clear in the water-bed experiment, where the concentric ring-wave pattern rotated the ratchet. This may be regarded as a spontaneous breaking of spatial symmetry in motion. In a previous experiment, a low frequency and amplitude were applied to the water bed and an ordered circular pattern appeared on the water surface^46^. However, this ordered pattern was violated at higher frequencies and amplitudes. Then, the surface pattern became more complicated, resulting in nearly random motion. In this situation, obtaining one-way spin using a geometrical ratchet mechanism is difficult. Herein, we present a novel ratchet mechanism based on the asymmetry of surface wettability, and this gear is geometrically symmetric. Studies of self-propelled droplets utilizing asymmetric wettability are reported^39,40,47–49^. These results suggest that asymmetric surface wettability may generate a directed driving force from noisy agitation caused by complicated surface waves. The obtained motion in the present study is a one-way spin for a longer timescale, but fluctuates in both directions for a shorter timescale. This newly proposed ratchet operates with asymmetry in the surface energy (wettability) and the periodically varying motion of the water surface. This may be closer to the well-discussed Brownian ratchet. To the best of our knowledge, this study presents a new ratchet structure that provides stochastic bias to random agitations.

## Results

**Figure 1:**
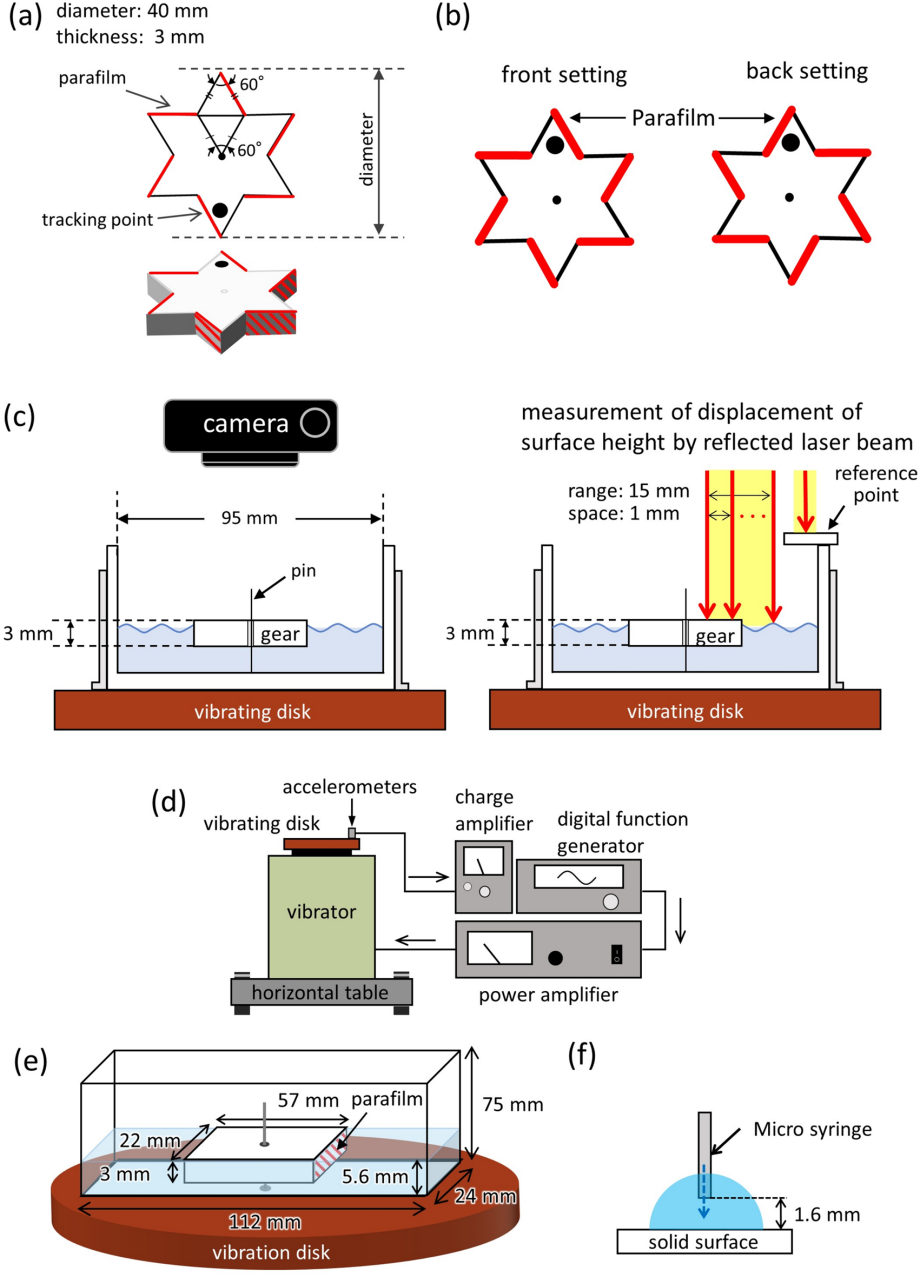
Experimental setup: (**a**) geometrically symmetric gear with a parafilm coating, (**b**) definition of front and back setting, (**c**) schematic of video recording and surface-height-profile analysis, (**d**) configuration of mechanical vibrator, (**e**) rectangular float on water, and (**f**) dynamic contact-angle measurement.

The schematics of the gear and experimental setup are shown in Fig. 1. On the side face of the symmetric gear, parafilm was attached alternately, as shown in red in Fig. 1a,b. The gear was geometrically symmetric and was asymmetric with respect to the upside-down inversion. In this study, the front setting was defined as that shown in Fig. 1b(left) when viewing the gear on the water from the top. The parafilm was attached to the right side of each of the six edges. The back setting is shown in Fig. 1b(right). The gear was made of acrylonitrile butadiene styrene (ABS) resin. The gear was placed on a water-filled Petri dish and was fixed with a push pin through the drilled hole. The petri-dish was placed on a vibrating disk that oscillated vertically at a predetermined frequency and amplitude (Fig. 1c).

### Rotation of gear

**Figure 2:**
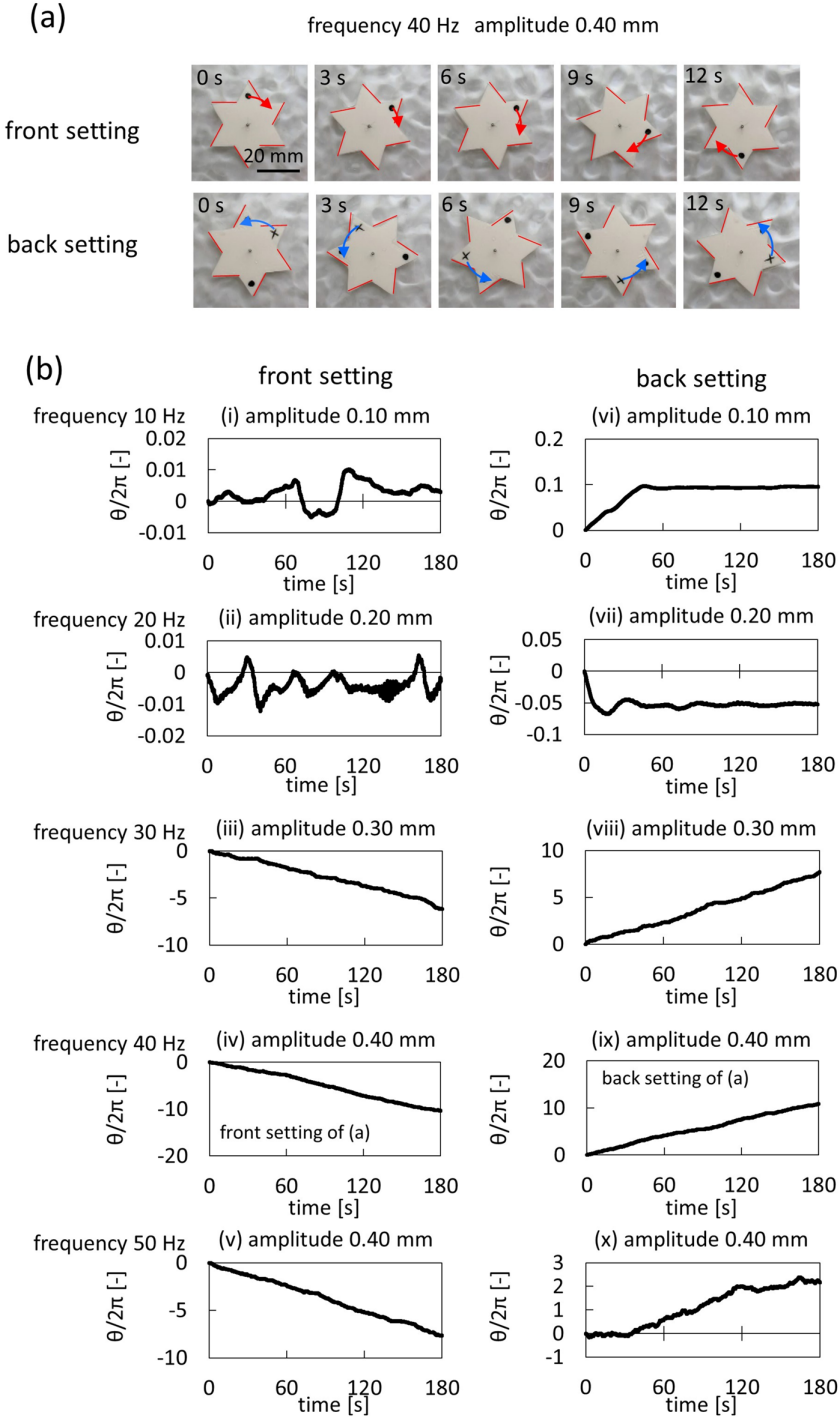
Results of spinning gear. (**a**) Snapshots of the spinning gear for front and back settings with vibrating frequency of 40 Hz and amplitude of 0.40 mm. (**b**) Cumulative rotational angle divided by 2π at each frequency and amplitude.

When the gear without parafilm was placed on the water bed with vertical oscillation, it did not exhibit a one-way spin for any frequency or amplitude employed in this study. Only fluctuational motion in both spinning directions (clockwise and anticlockwise, shown in the Supplementary Movie) was observed. In contrast, when a gear with parafilm (Fig. 1a,b) was placed on the water bed, the gear exhibited a one-way spin in a restricted range of the frequency and amplitude. The time course of the snapshots are exemplified in Fig. 2a. When the gear exhibited one-way spin, the spin direction was determined by the chirality of the gear, and the spinning direction was opposite between the front and back settings (Fig. 1b). This is shown in Fig. 2a, which indicates that the side face with parafilm was at the front and that without parafilm was at the rear (the red line (Fig. 2a) in the photographs indicates the parafilm side). Therefore, when the gear was in the front setting (Fig. 1b), the spin direction was clockwise from the top view, and vice versa.

The rotational angle around the center of the gear was measured as a function of time. The cumulative rotational angle (divided by 2π) at each frequency (*f*) and amplitude (*A*) are shown in Fig. 2b. The results were extracted from experiments conducted over a wide range of amplitudes and frequencies. At the lower frequencies and amplitudes such as *f* = 10 and 20 Hz and *A* = 0.10 and 0.20 mm, the gear showed fluctuating rotation with both spinning directions (clockwise and anticlockwise) and did not exhibit a stable one-way spin. In contrast, at higher frequencies (30, 40, and 50 Hz) and larger amplitudes (0.30 and 0.40 mm), the gear exhibited a one-way spin when observed with a longer timescale (at a shorter timescale, the angular velocity fluctuated, which will be discussed later). For the front setting (back setting), the cumulative rotational angle was negative (positive), which represented clockwise (anticlockwise) spin. The comparison of the result with constant amplitude and varying frequencies or vice versa are shown in SI (Fig. S1). The experiments were repeated more than five times, and similar results were obtained.

**Figure 3:**
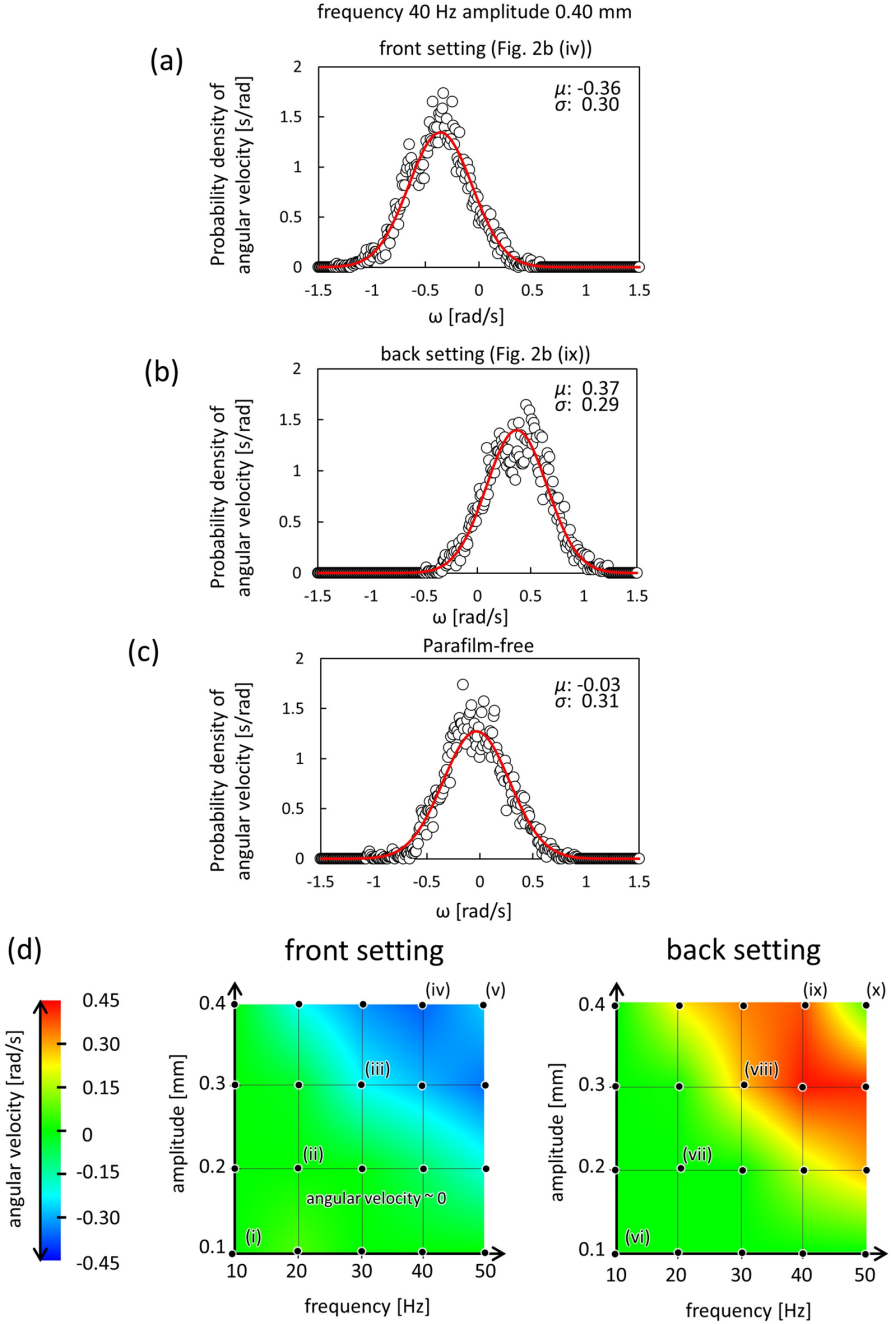
The characteristic of the gear motion. The probability density distribution of the angular velocity is calculated for the result of Fig. 2b(iv) (**a**), Fig. 2b(ix) (**b**), and the parafilm-free experiment (**c**) at *f* = 40 Hz and *A* = 0.40 mm. The angular velocity is the average for 1 s. The red curve on the figure shows the Gaussian distribution with the mean value (*μ* [rad/s]) and dispersion (*σ* [rad/s]) shown in the figure. (**d**) Average angular velocity for 180 s is shown on *f*-*A* plane. The angular velocity is measured every 10 Hz (*f*) and 0.1 mm (*A*) and is shown by a color map with the appropriate interpolation (GRAVITIC mode, RINEARN). The scale (color) bar is indicated on the left. The data with the numbering (i–x) are those used for Fig. 2b.

Figure 3 shows the probability density distribution of the angular velocity (*ω*) measured every 1 s (the shorter timescale). These results were calculated from those of Fig. 2b (iv) and (ix) and of the parafilm-free gear at *f* = 40 Hz and *A* = 0.40 mm (SI). The distribution of the angular velocity spread over positive and negative values for all the results, including those of the parafilm-coated gear. This indicates that the angular velocity fluctuated for a shorter timescale in both directions. However, the cumulative angles shown in Fig. 2b (iv) and (ix) exhibit a one-way spin for a longer timescale. The red curves on the figure show a Gaussian distribution with its mean value (*μ* [rad/s]) and dispersion (*σ* [rad/s]). The experimental results were approximately correlated with the Gaussian distribution. These results suggest that the elementary motion followed a random-walk process and that the parafilm-coating caused a biased probability in the random walk. The spin caused by the random walk process was essentially different from that with the geometrically asymmetric gear^46^ at lower frequencies and amplitudes, where the driving force was deterministic and did not cause outstanding fluctuations in the spinning directions. The driving force for the gear of this study was not deterministic but stochastic.

Angular velocities averaged over 180 s (longer timescale) are shown in Fig. 3d. (Ordinary graphic presentation for typical results are shown in SI (Fig. S2)) The angular velocity was measured every 10 Hz (*f*) and 0.1 mm (*A*) and shown on the *f*-*A* plane via a color map with the appropriate interpolation (GRAVITIC mode, RINEARN) (this method is shown in the SI (Fig. S3)). The blue and red domains show the clockwise ($\omega <0$) and anti-clockwise ($\omega >0$) spins, respectively. In the green domain, the average angular velocity was nearly zero. The color pattern was almost symmetrically reversed between the front and back settings. This indicates that the angular velocity was determined by the chirality. For both the front (left) and back (right) settings, a larger absolute value of the angular velocity, which corresponds to a stable one-way spin, appeared in a higher frequency and amplitude area. At lower frequencies and amplitudes, the results indicated that the gear did not exhibit stable one-way spin.

### Dynamical pattern of water surface

**Figure 4:**
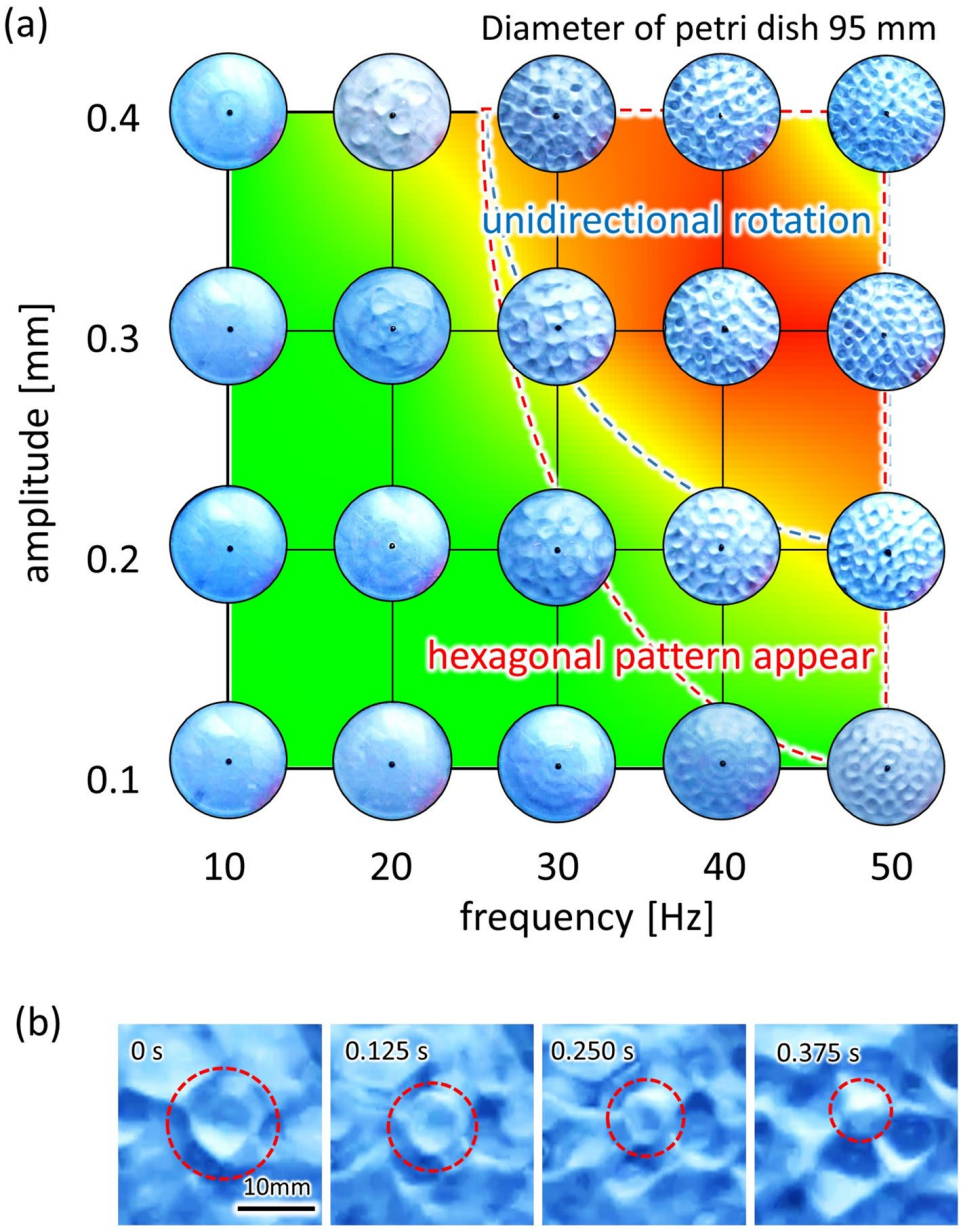
The surface wave patterns. (**a**) Top view of the vibrating water-filled petri dish with a diameter of 95 mm. The photographs are placed at each frequency and amplitude on the *f*-*A* plane of Fig. 4a (back setting). The surface-wave pattern showed a honeycomb-like pattern on the right side of the red dashed curve, whereas the concentric-ring pattern appeared on the left side. The region that provided the one-way spin is positioned on the right side of the blue dashed curve. (**b**) The time-course of a convection cell at* f* = 40 Hz and *A* = 0.40 mm. The cell encircled by red periodically spreads and shrinks along the radial direction.

Figure 4a shows the surface patterns of the oscillating water surface at each frequency and amplitude. Typical snapshots are overlaid in Fig. 3d (back setting). The water surface showed a honeycomb-like pattern on the right side of the red dashed curve. The honeycomb-like pattern reflected the Faraday-wave formation^50^. This was confirmed by a frequency analysis of the oscillating water surface, where the oscillating frequency was half that of the external vibration (SI, Fig. S3). In contrast, a concentric-ring pattern appeared on the left side of the red dashed curve. The region with the larger angular velocity was positioned on the right side of the red dashed curve, indicating that the one-way spin required a honeycomb-like surface pattern. Figure 4b shows the time course of the surface pattern at a frequency of 40 Hz and an amplitude 0.40 mm. The cell enclosed by the red dashed circle oscillates, and each honeycomb-like cell periodically spreads and shrinks along the radial direction.

**Figure 5:**
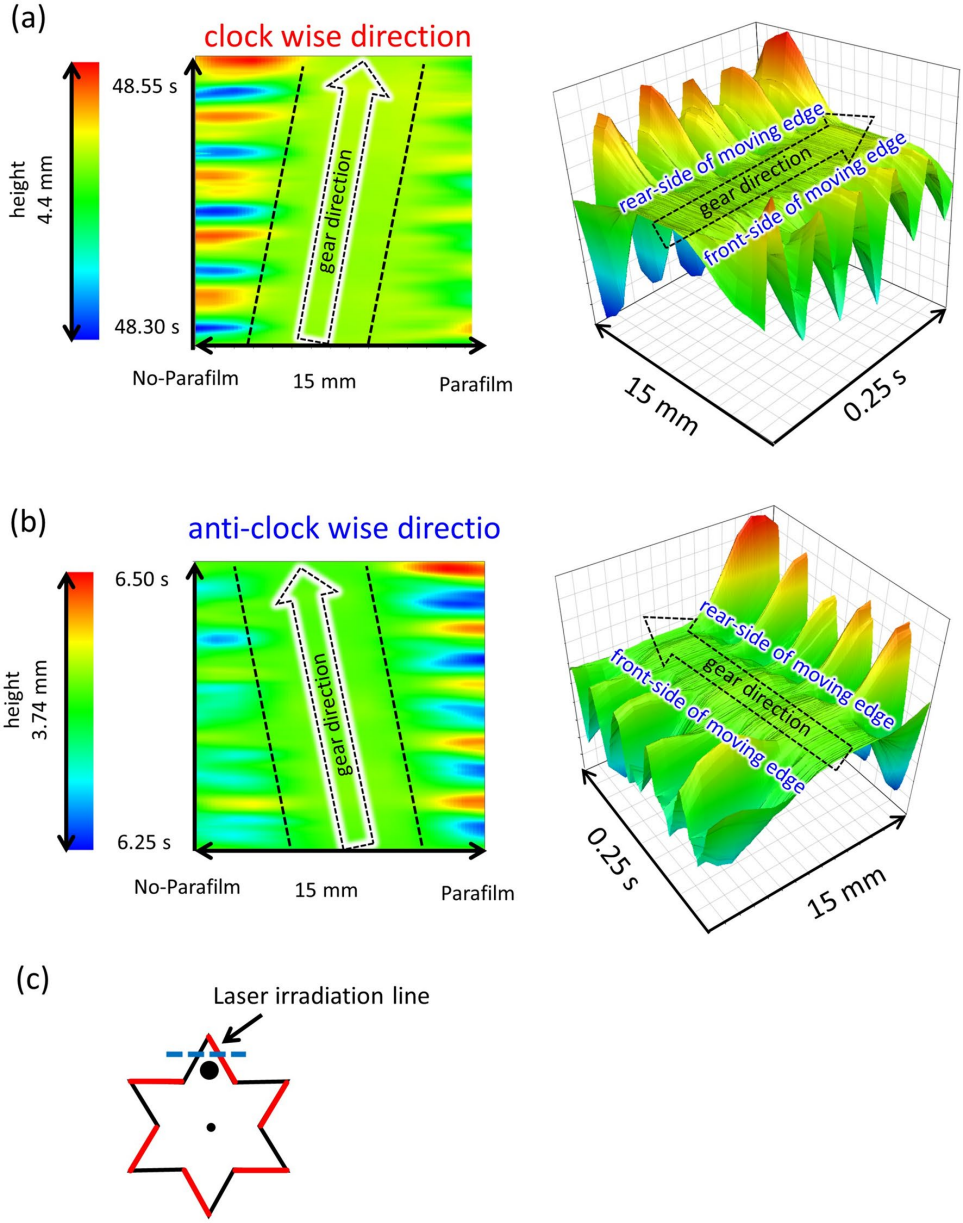
Space–time plot of the height profile along the laser-irradiated line shown in (c) at *f* = 40 Hz and *A* = 0.40 mm. These are measured by the laser displacement meter that detects the height every 1 mm over 15 mm length. The time course of the height profile is piled from the bottom to the upper direction, and the resultant space–time plot is represented by color. The direction of the gear rotation depends on the time range in an experiment, as shown in the angular-velocity distribution, e.g., Fig. 3a and b. The rotation direction is (a) clockwise and (b) anticlockwise. Note that these are the results for the same experiment with the same gear for different time ranges. The 3D graph shows a bird’s-eye view of the space–time plot to visualize the height of the meniscus rise at the side face of the gear.

Figure 5a,b show the space–time plot of the height profile along the laser-irradiated line shown in Fig. 5c. These were measured using a laser displacement meter that detected the height every 1 mm over a 15 mm length. Data acquisition was performed at 10^3^ Hz over 0.25 s. The time course of the height profile was piled up from the bottom to the upper direction, and the resultant space–time plot was represented by color. The space–time plot of the experiment with *f* = 40 Hz and *A* = 0.40 mm is shown. In this experiment, the gear exhibited a stable one-way spin over a longer timescale. However, as shown in Fig. 3a–c, the spinning direction fluctuated within a shorter timescale, such as 1 s. Because the duration of the space–time plot was 0.25 s, the spinning direction depended on the extracted timespan. The typical results corresponding to both spinning directions are presented in Fig. 5a,b; the results are of the same experiment with the same gear at different time ranges. The gear top (surface) corresponds to the area between the two black dashed lines shown in the figure. In Fig. 5a, the gear top moves from left to right with time (the direction is indicated by the arrow). This corresponds to the clockwise spin of the gears. In contrast, the spin direction is anticlockwise in Fig. 5b. The 3D graphic on the right side of the figures shows the dynamic motion of the meniscus on the side face of the gear. In both cases, the amplitude of the oscillating water surface was larger at the rear of the moving gear, where the color oscillated periodically between blue and red. At the front of the moving gear, the amplitude was smaller, as indicated by the greenish color.

### Surface wettability

**Figure 6:**
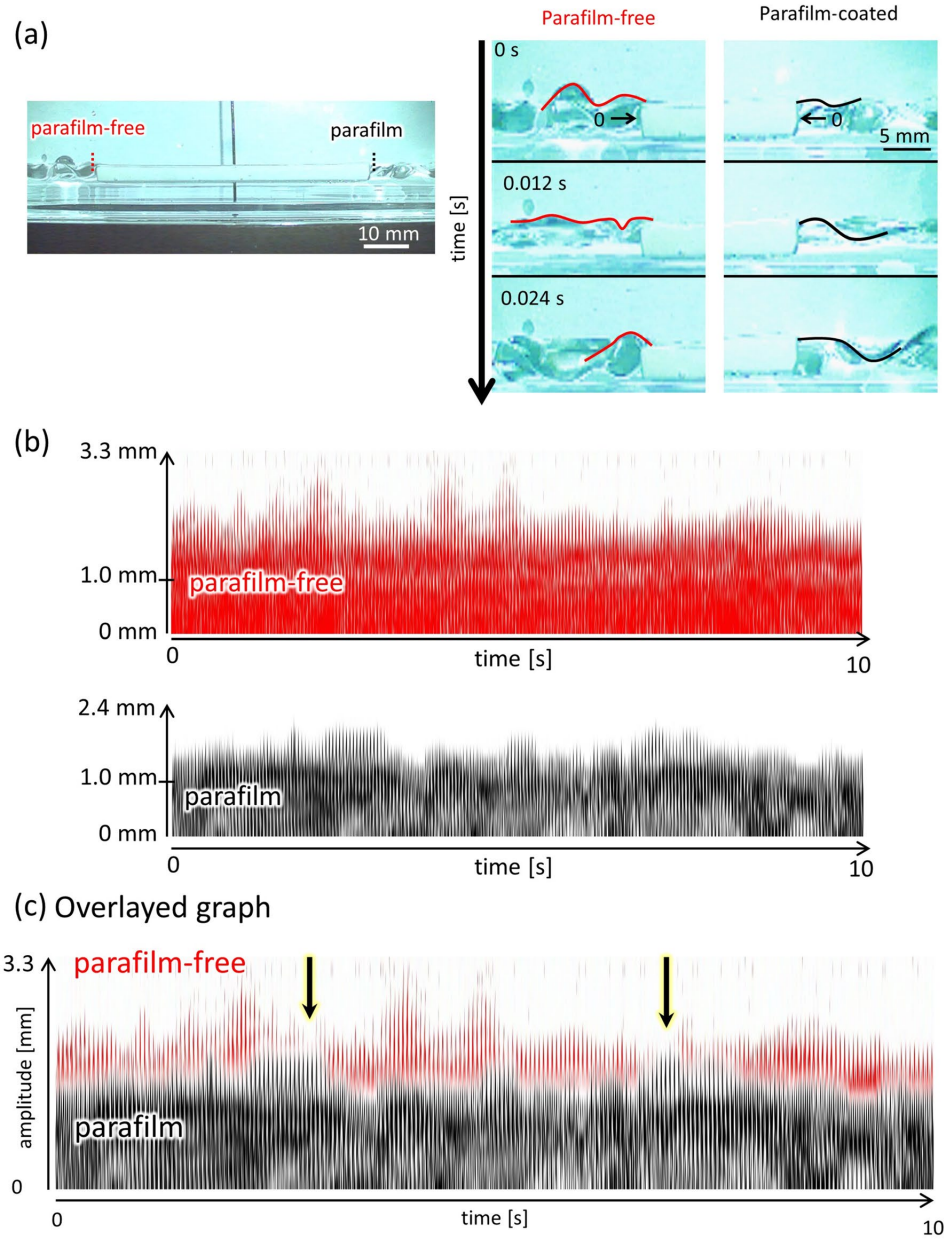
Meniscus motion in the vicinity of the sidewall of the float. The rectangular-shaped cell with water is vibrated at *f* = 40 Hz and *A* = 0.40 mm (**a**) Snapshot of the meniscus motion. The red and black dotted lines denote the parafilm-free and -coated sides, respectively. (**b**) The space–time plot is composed of these lines (**a**). It is binarized and colored red for parafilm-free and black for parafilm-coated sides. The zero value of ordinate is indicated in Fig. 6a (right) (**c**) The black space–time plot was overlapped on the red plot to compare the amplitudes. In most cases, the red amplitude is larger than the black one; however, this is sometimes reversed.

The rectangular float was placed on the water as shown in Fig. 1e. The parafilm was attached to one of the sidewalls. This experiment was performed considering the effect of the parafilm on the meniscus motion. A rectangular cell filled with water was vibrated at *f* = 40 Hz, *A* = 0.40 mm. Snapshots of the meniscus motion are shown in Fig. 6a. These snapshots were extracted from the typically observed motion. The amplitude in the vicinity of the sidewall was larger on the parafilm-free side, and vice versa. Note that this was a typical result, and a case in which this trend is unclear also exists. The space–time plot shown in Fig. 6b was obtained from the vertical dotted lines shown in Fig. 6a. The space–time plot was binarized and colored. Black and red corresponded to the parafilm-coated and parafilm-free sides, respectively. The black plot overlapped the red plot (Fig. 6c). In most cases, the amplitude in red was larger than that in black. However, an exceptional case was occasionally observed, as indicated by the arrows. The results showed that the wave amplitude was stochastically larger on the parafilm-free side.

**Figure 7:**
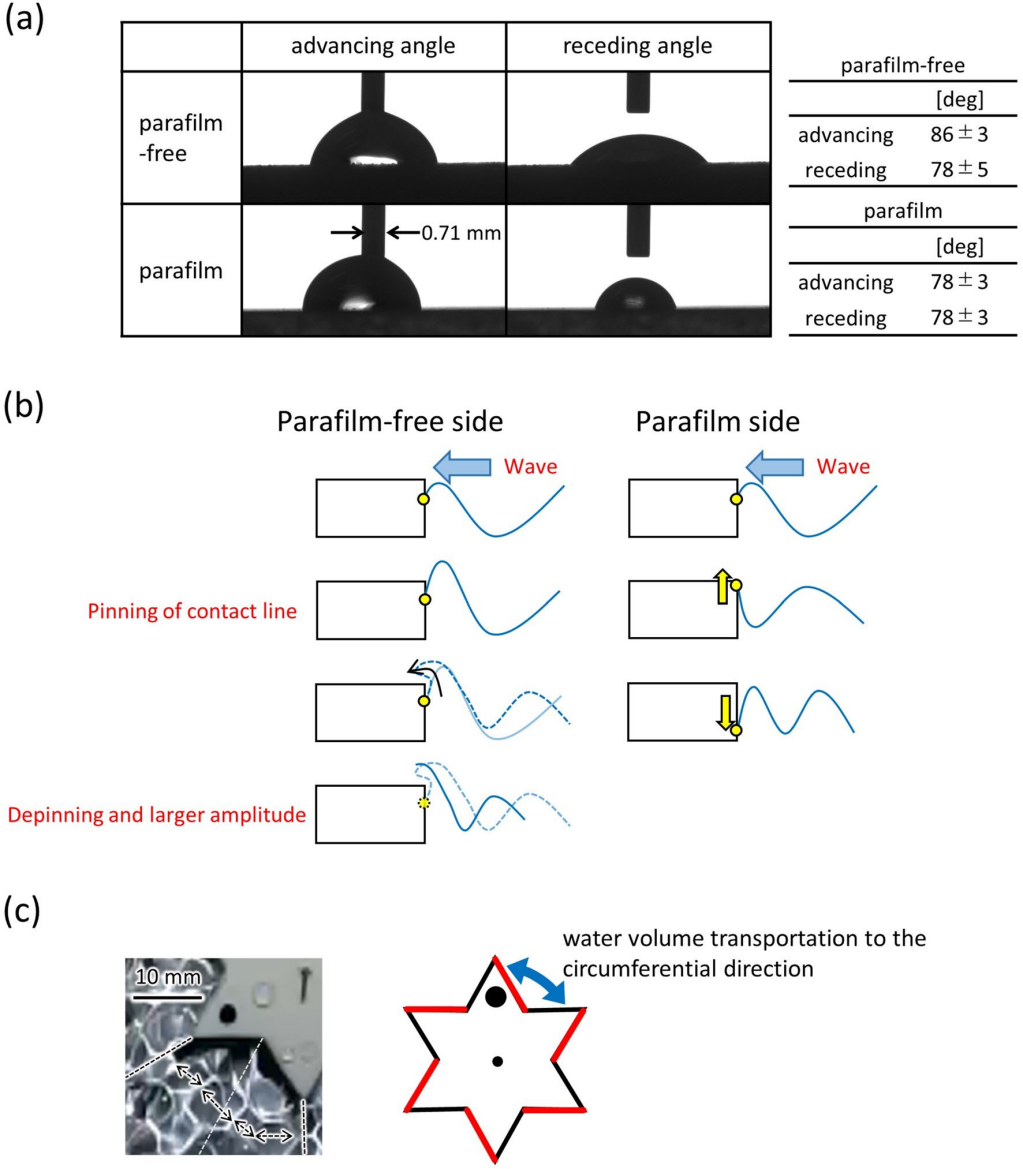
The effect of surface wettability. (**a**) Photograph of the contact-angle measurement on the substrate made of ABS with and without parafilm. The table beside the photograph shows the advancing and receding contact angle at each surface. (**b**) Schematic representation of the meniscus motion. At the parafilm-free side, the gear surface tends to trap the contact line, and then the rising meniscus is trapped. In this case, once this pinning is released, the amplitude of the meniscus rise increases. Therefore, the contact line drastically slides up and down. In contrast, on the parafilm-coated side, the contact-line motion becomes smoother; hence, the meniscus rise is not amplified. (**c**) Snapshot of the water surface between the adjacent gear teeth and a schematic of the direction of the water-volume transport.

The dynamic contact angle of the water on the substrate was measured using a sessile drop experiment shown in Fig. 1f. A snapshot of the contact angles on the ABS substrate with and without the parafilm is shown in Fig. 7a. The table adjacent to the photograph shows the advancing and receding contact angles on each surface. The hysteresis between the advancing and receding contact angles was larger for the parafilm-free surface. The Parafilm-free surface was slightly messy, although it was polished as a gear surface. This messy surface tended to trap the contact-line motion. In contrast, the surface with parafilm was very smooth, which was responsible for the smaller hysteresis. When the gear surface without parafilm trapped the contact line, the rising meniscus at the sidewall was also trapped. Once this pinning was released, the wave amplitude increased, as schematically shown in Fig. 7b. In this case, the contact line slid up and down drastically on the parafilm-free side. In contrast, on the parafilm-coated side, the contact-line motion became smoother; hence, the amplitude was not amplified.

When the wave crest rippled, the water volume contained in the wave crest was forced to move to the neighboring crest. This horizontal transport of water provided a pushing force on the side face of the gear^46^. As shown in Figs. 4 and 7c, the surface-wave pattern is complicated, and the size of each convection cell is as small as the distance between adjacent gear edges. In this case, the horizontal transport of water volume between the gear edges may occur along the circumferential and radial directions. As shown in Fig. 6, the amplitude of the contact-line motion was stochastically larger (smaller) on the parafilm-free (-coated) side. Then, the force to push the side face was stochastically larger at a larger amplitude, that is, on the parafilm-free side. Therefore, the force generated by this water transport was stochastically unbalanced between the two sides of the gear edge. Because this was a stochastic process, the spinning direction fluctuated over a shorter timescale. However, the angular velocity averaged over a longer timescale was determined such that the gear face without the parafilm became the rear side. A stochastic bias in the driving force must be produced by the effect of the parafilm on meniscus motion.

Following the mechanism discussed above, the one-way spin is expected to appear effectively when the size of unit convection cell is as small as or smaller than the distance between the adjacent gear edges. However, the mechanism could not work well when the convection cell is too small, because it requires the larger frequency and amplitude. Then, the deformation of water surface becomes more violent that will be hardly controlled by the surface wettability. Therefore, the optimum frequency and amplitude for the average angular velocity appears at their intermediate ranges. This is seen in Fig. 3d. (This is clearer for back setting result.)

### Random walk model

**Figure 8:**
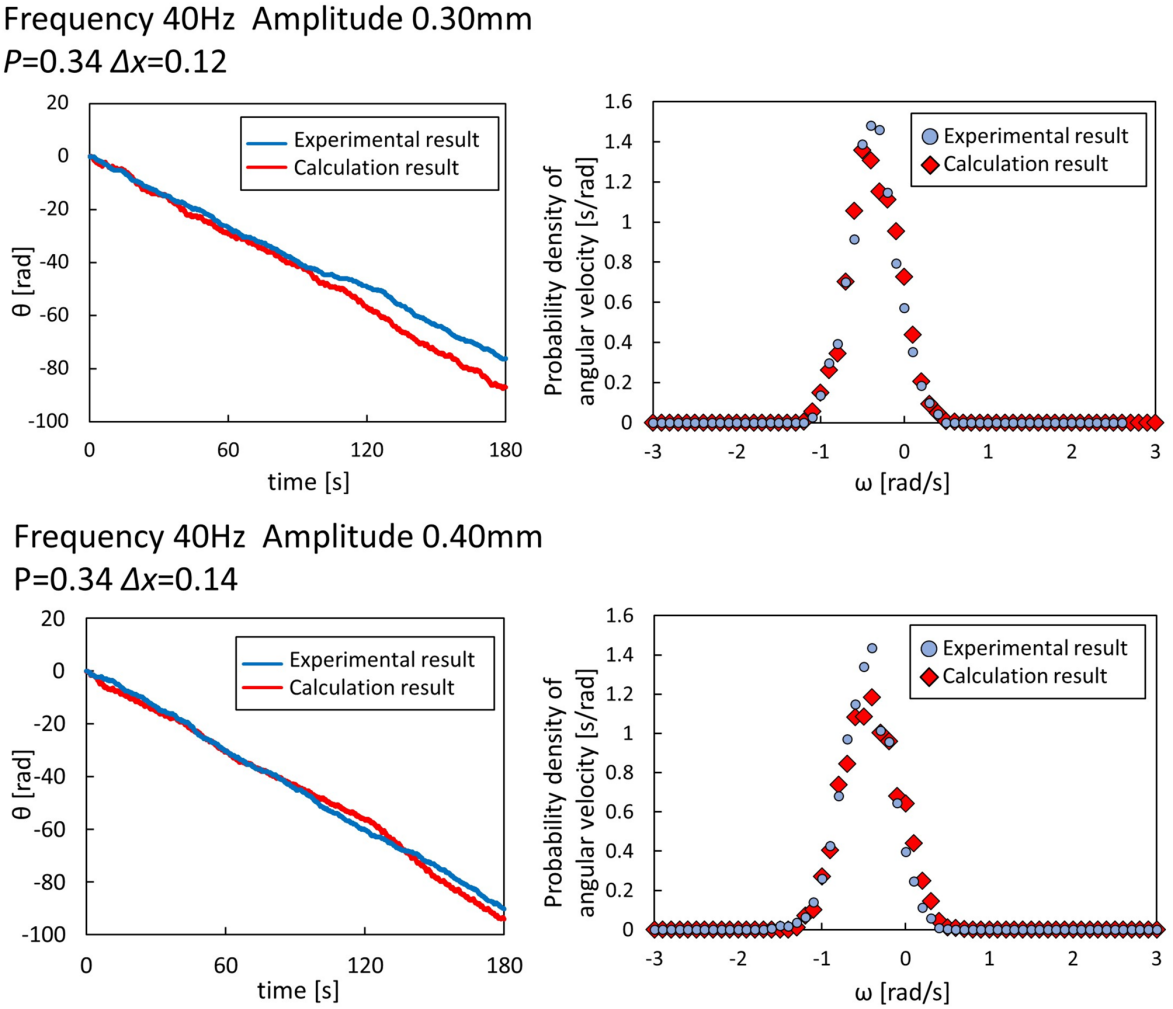
Calculation result of the biased random-walk model. The characteristic of the gear motion is explained by a random-walk model with a biased probability (*p* and 1-*p*) and elementary step distance (*Δx*). The calculation results are overlaid on the experimental results obtained for the cumulative angle and probability density distribution of the angular velocity at* f* = 40 Hz and* A* = 0.30, and 0.40 mm. The fitting parameter *p* and *Δx* are mainly determined by the results of the dispersion of the distribution and by the cumulative angle (mean value of distribution), respectively. Red and blue correspond with the calculated and experimental results, respectively. The angular velocity is the average over 1 s for the experimental and calculated results.

The results of Fig. 6 indicate that the amplitude of parafilm-free side is dominantly larger. This suggests that the gear spins could be deterministic. However, the angular velocity shown in Fig. 3 exhibits notable stochastic characteristic in the spinning motion. Here, the stochastic nature of the gear motion is discussed semi-quantitatively by a random-walk model with a biased probability. A one-dimensional random-walk model was adopted. The mean square displacement was determined by the number of steps under the postulated values of a bias in the probability (*p* and 1−*p*) and elementary step distance (*Δx*). The calculation procedure is written in SI. The elementary motion was assumed to occur every 0.05 s because the frequency of the surface wave was half of the external vibration frequency (40 Hz). Faraday waves were developed on the water surface, and the relationship between the two frequencies was confirmed experimentally. The cumulative angles (Fig. 2b) and probability-density distribution of the angular velocity (Fig. 3a) could be reproduced by the random-walk model with two adjustable parameters, *p* and *Δx*. To reproduce both the cumulative angle and angular-velocity distribution, the fitting parameter was required to be *p* $\approx$ 0.40, and *Δx* [rad] was dependent on the result of the cumulative angle. The calculation results of the random-walk model are shown in Fig. 8. The probability *p* was determined not to be far from 0.5. This indicates that the stochastic nature of the driving forces is significant. Based on Fig. 6b, the amplitude on the parafilm-free side was dominantly larger than that on the parafilm-coated side, suggesting that the probability *p* was more apart from 0.5. The discrepancy in *p*-values between the experiment and the calculation is probably because the adjacent gear teeth were so close that the meniscus motions of the adjacent sidewalls affected each other through the water surface. This interaction caused the levelling of the meniscus motions to prevent an imbalance in the biased probability.

## Discussion

A geometrically symmetric gear with chirality made using parafilm coating exhibited a one-way spin on the vibrating water bed at higher frequencies and amplitudes. The angular velocity fluctuated in both directions for a shorter timescale; however, for a longer timescale, the gear exhibited one-way spin. The side face with the parafilm became the front, and that without parafilm became the rear for the one-way spin. This unique motion was induced by a stochastic process with a biased driving force produced by the interaction between the vibrating water surface and side face of the gear. The meniscus motion at the side face was greater on the parafilm-free side, and the oscillating water pushed this side. The hysteresis between the advancing and receding contact angles was larger on the parafilm-free surface. The messy surface tended to trap the contact-line motion, resulting in the pinning of the contact line followed by a large rebound after depinning. In contrast, the surface with parafilm was very smooth, which was responsible for the smaller amplitude of the contact-line motion. The imbalance in the meniscus motions became stochastic through the interaction of the contact-line motion at the adjacent menisci.

The ratchet surface potential, that is, the asymmetry of the surface wettability, caused spin, where a cyclic wave motion was transformed into a regulated spin. This ratchet-motor system was significantly different from a geometrically asymmetric ratchet driven by a deterministic mechanism. Considering that a molecular ratchet works on an asymmetric potential with cyclic variation, the concept inherent in this system may provide a breakthrough in generating a new ratchet-motor design. We believe that this study expands the field of ratchet motors and provides intriguing ideas for physicochemical ratchets on a smaller scale.

## Methods

The schematics of the gear and experimental setup are shown in Fig. 1. On the side face of the symmetric gear, parafilm (PM992, Bemis Flexible Packaging) was attached alternately, as shown in red in Fig. 1a,b. The gear was geometrically symmetric and was asymmetric with respect to the upside-down inversion. In this study, the front setting was defined as that shown in Fig. 1b(left) when viewing the gear on the water from the top. The parafilm was attached to the right side of each of the six edges. The back setting is shown in Fig. 1b(right). The gear diameter, thickness, and weight were 40 mm, 3 mm, and 1.47 g, respectively. The gear was made of acrylonitrile butadiene styrene (ABS) resin and fabricated using a three dimensional (3D) printer (1.0 pro 3-in-1, XYZ-Printing). The gear surface was polished with sandpaper to obtain a smooth surface, and a hole with a diameter of approximately 1 mm was drilled at the center.

The gear was placed on a water-filled Petri dish with a diameter of 95 mm and height of 30 mm, as shown in Fig. 1c. Fully deionized water (ELGA Labwater, Purelab Flex 3; 40 mL) was poured into the Petri dish placed on the vibration disk. The water depth was 5.6 mm. The gear was fixed with a push pin through the drilled hole, such that it was placed at the center of the Petri dish. The push pin was attached vertically to the bottom of the Petri dish.

The setup of the vibrator (513-B, EMIC Co.) is shown in Fig. 1d. The vibrator was connected to a digital function generator (DF1906, NF Co.) that generated a sinusoidal wave, and an accelerometer with a charge amplifier (505-CBP, EMIC Co.) was attached to the vibrating disk. The amplitude was controlled using a power amplifier (371-A/G, EMIC Co.). The vibrating disk, in which the Petri dish was fixed, oscillated vertically at a predetermined frequency and amplitude.

The motion of the gear was monitored using a digital camera (EX-100F, CASIO). The position of a fixed point at the tip of the gear was traced using TEMA (Photron). The azimuth of the tracking point on the gear was measured and used to calculate the angular velocity.

The changes in the height profiles of the water surface and gear top were measured using a laser displacement meter (LJ-V7080, Keyence). The height profile was measured every 1 mm over a 15-mm range at a 10^3^ Hz data-acquisition rate. The measured height was the distance between the measured surface and a reference point moving with the vibrating substrate, as shown in Fig. 1c(right). Thus, the time course of the height was the change in the vertical position of the surface relative to that of the vibrating substrate. When a laser displacement meter was used, a small amount of white paint was mixed with water to enhance reflection. The effect of paint on the gear dynamics was negligible. The 3D figures and space–time plots were obtained using free software (RINEARN Graph 3D, Ver. 5.6.26, Fumihiro Matsui, Japan, https://www.rinearn.com/en-us/).

Another experiment with a rectangular cell was performed to observe the meniscus motion at the sidewall of a float. A schematic of the float and experimental setup are shown in Fig. 1e. The float was created using the same method employed for the gears. The lengths of the longer and shorter sides were 57 and 22 mm, respectively, and the thickness was 3 mm, which was identical to the thickness of the gear. A hole with a diameter of approximately 1 mm was drilled at the center. The surface of the float was polished with sandpaper, and parafilm was attached to one of the sidewalls, including the shorter length (22 mm). The dimensions of the rectangular cell were 112 mm (longer-side), 24 mm (shorter-side), and 75 mm (height) (Fig. 1e). Fully deionized water (15.05 mL) was poured into the rectangular cell. The depth of the water was 5.6 mm, which was the same as that used for the Petri-dish experiment. The float was placed on the water and at the center of the rectangular cell. The float was fixed with a push pin inserted through the drilled hole, which was attached vertically to the bottom of the cell. A motion picture of the moving meniscus was captured from the side against the longer axis of the rectangular cell. Videos were captured using a motion-analysis microscope (VW-6000, KEYENCE) at 500 fps. A space–time plot was obtained using the open-source software ImageJ (ver. 1.53 k 6 July 2021, National Institute of Health, https://imagej.net/ij/).

The dynamic contact angle of the water on the substrate was measured using a sessile drop experiment. The experimental setup is illustrated in Fig. 1f. A water droplet was placed using a micro syringe (Hamilton; 1000 *μ*L) by syringe pump (PHD Ultra, Harvard Apparatus) on the substrate made of ABS with and without the parafilm coating. The infuse/withdraw speed was 2 *μ*L/s. The droplet shape was captured using an optical tensiometer (Theta, KSV Instruments LTD) to measure the contact angle using ImageJ (Drop Analysis-Drop snake). The advancing and receding contact angles were measured by manipulating the syringe pump.

### Supplementary Information

Supplementary Information 1.

Supplementary Movie 1.

## Data Availability

The datasets used and/or analyzed during the current study available from the corresponding author on reasonable request.
