# Supplementary material for: Surface-energy ratchet motor with geometrical symmetry driven by biased random walk
Source: Sci Rep. 2024 Jul 18;14:16619. doi: 10.1038/s41598-024-67383-1 (PMC11258250; doi:10.1038/s41598-024-67383-1)
Supplement: Supplementary file 1 — Supplementary Information 1. [file 41598_2024_67383_MOESM1_ESM.pdf]

SI

Surface-energy ratchet motor with geometrical symmetry driven by biased random walk

Miku Hatatani, Daigo Yamamoto, and Akihisa Shioi*

Department of Chemical Engineering and Materials Science, Doshisha University
1-3 Tatara Miyakodani, Kyotanabe, Kyoto 610-0321 Japan
E-mail: ashioi@mail.doshisha.ac.jp

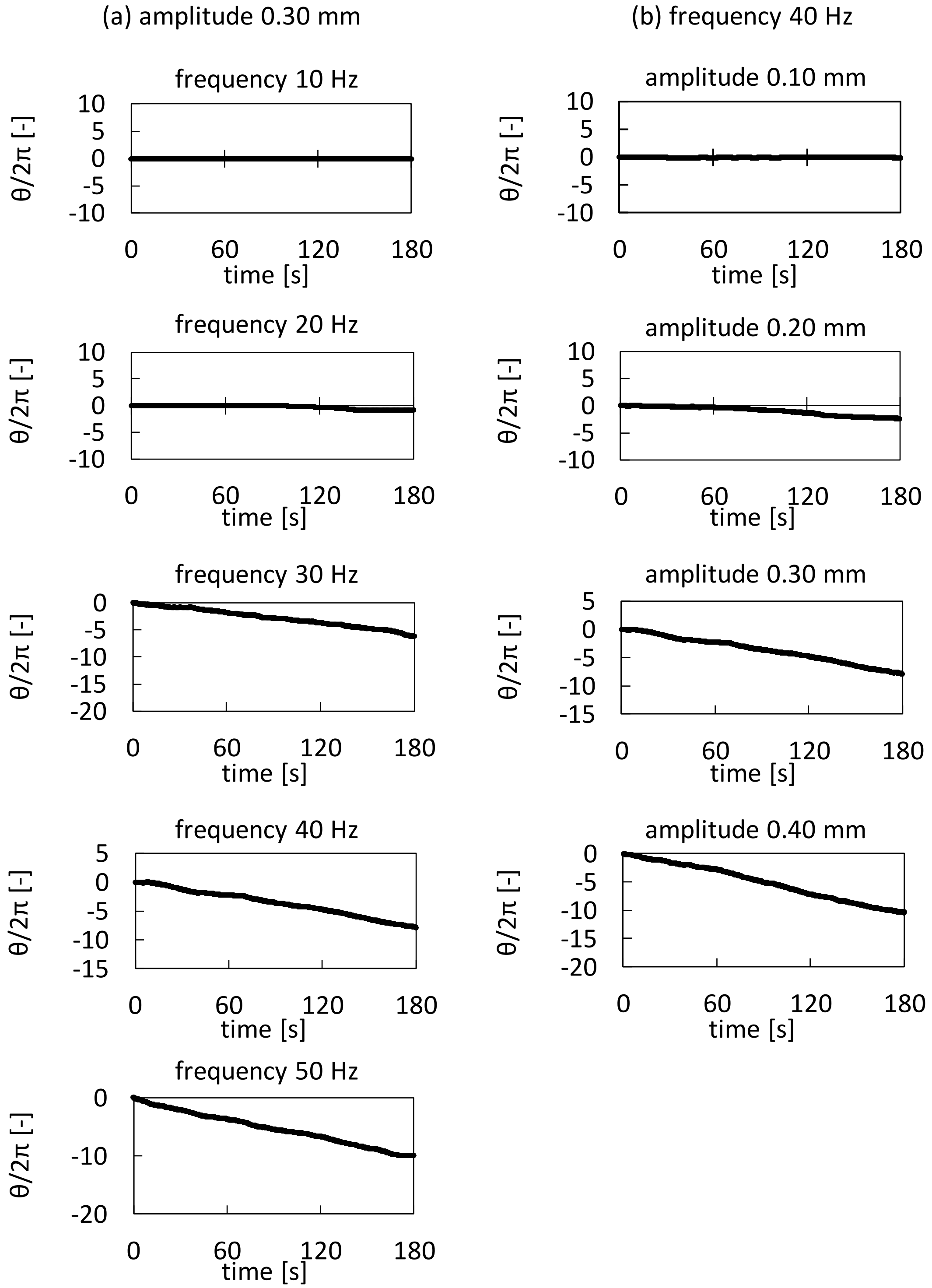

Results of cumulative rotational angle divided by 2π. (a) Vibration amplitude 0.30 mm. (b) vibration frequency 40Hz.

Figure S1

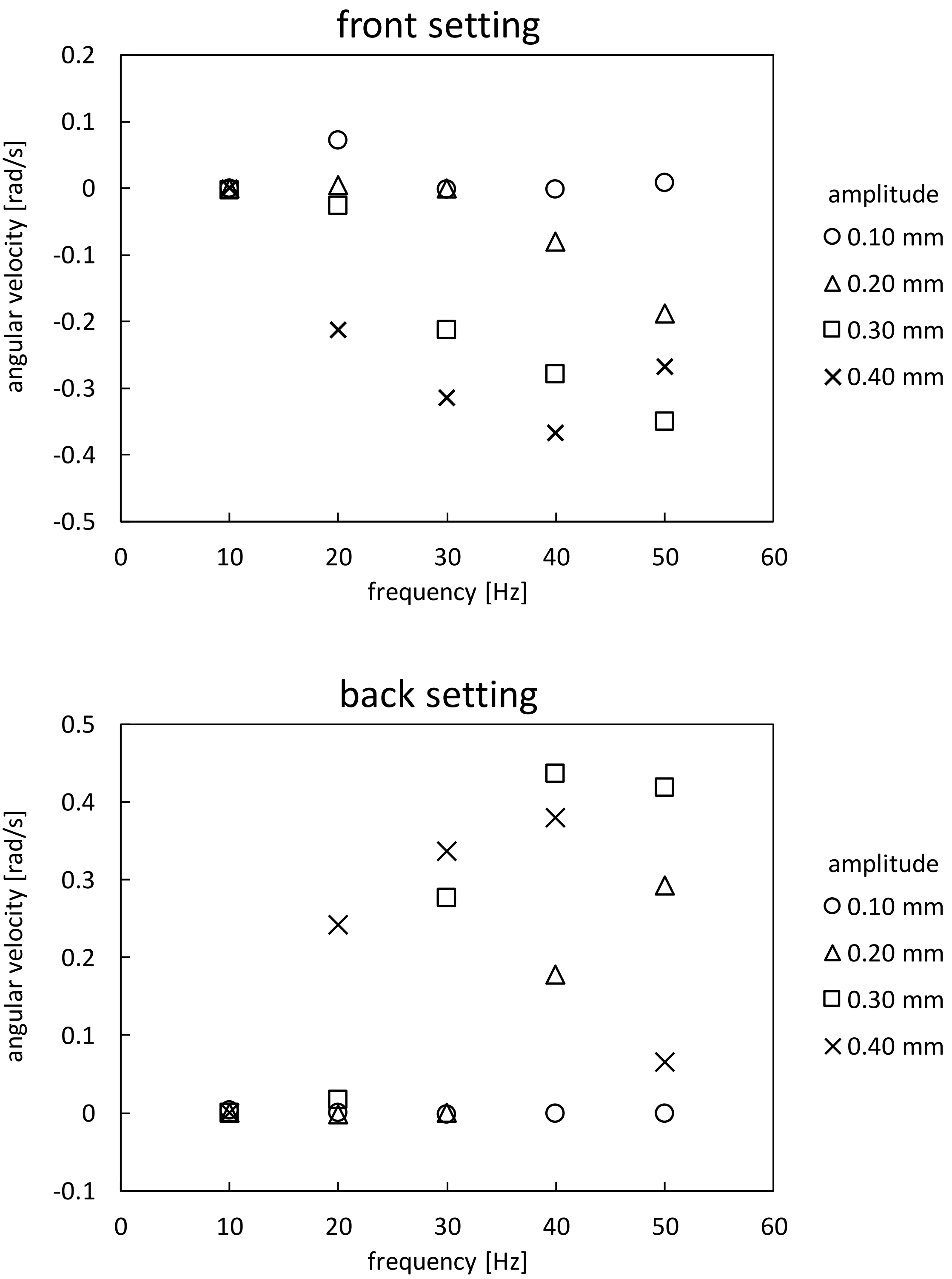

Results of angular velocity at each frequency and the amplitude. The different markers indicates the each vibration amplitude ○= 0.10, △= 0.20, □= 0.30, and ×= 0.40 mm.

Figure S2

“GRAVITIC” mode, Rinearn

The "GRAVITIC" mode completes the center of gravity of four adjacent points from the coordinate values, then completes the center of gravity of four adjacent points from there, and generates a surface obtained as the extreme limit of repeating this operation forever. The surface obtained by this completion faithfully traces the position of each coordinate point and preserves the original sharpness. It is suitable for data that should be highly uneven by nature.

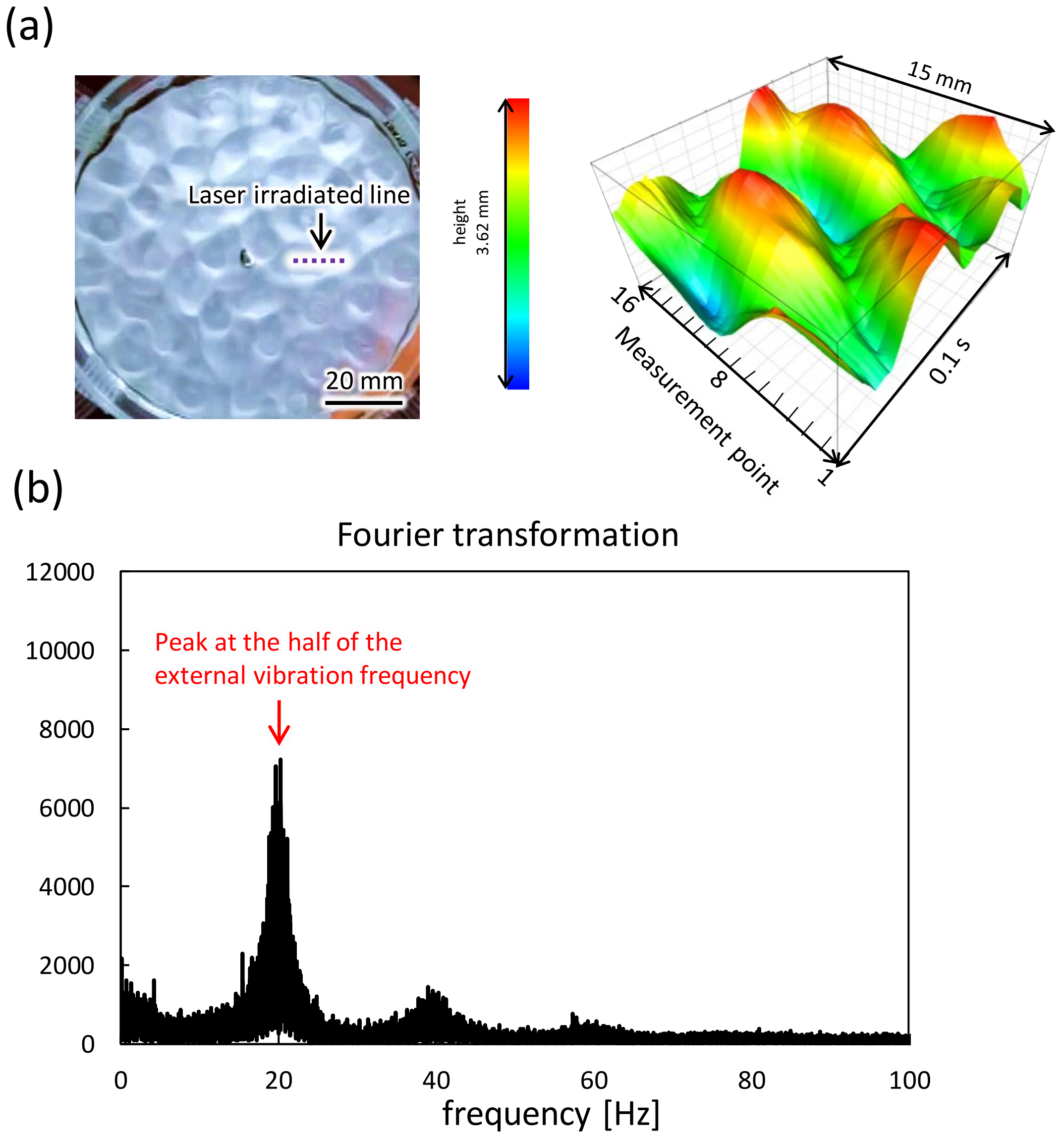

(a) The 3D space–time plot of the height profile along the laser-irradiated line and (b) the Fourier spectrum for 180 s at measurement point 8. The water bed was vibrated with f=40 Hz and A=0.40 mm. The oscillating frequency was the half of the external frequency, indicating the Faraday wave formation.

Figure S3

The calculation procedure of one-dimensional random-walk model

A moving point occupies a point on a number line (one dimensional). The coordinate of the occupied point at step i is $N_i r\Delta x_{max}$, where N_i is integer and Δx_{max} is a constant value with radian. r is the random number between 0 and 1. The point moves on the line step-by-step. Each step follows the rule:

$$
\begin{aligned}
N_{i+1} &= N_i + 1 \text{ at probability } p \\
&= N_i - 1 \text{ at probability } 1 - p
\end{aligned}
$$

There are two adjustable parameters, Δx_{max} and p. Δx_{max} depends on the impact of collision by water volume at the gear wall that is mainly determined by the surface-wave amplitude. This collision pushes the gear wall. In this model, the probability pushing the parafilm-side is p and 1-p for parafilm-free side. Experimental result suggests $p < 0.5$ (Figure 6). The surface wave is not so regulated, and hence the pushing force fluctuates. This effect is takes into account by the random number r. Then, Δx_{max} corresponds to the possible maximum pushing force, and the pushing force Δx (main text) is equal to $r\Delta x_{max}$. Each step is assumed to occur at periodicity Δt. In this calculation, we consider that Δt is equal to the reciprocal of the surface wave frequency. As the Faraday wave appears at water surface (Fig.S3), the surface wave frequency is taken as the half of the external frequency.
